# Reproductive evaluation of Luisa, the last jaguar of the Caatinga

**DOI:** 10.1590/1984-3143-AR2023-0090

**Published:** 2023-11-20

**Authors:** Letícia Alecho Requena, Thiago Cavalheri Luczinski, Anneliese de Souza Traldi, Thyara de Deco-Souza, Gediendson Ribeiro de Araújo, Cristiane Schilbach Pizzutto, Giovana Martins Miranda, Mirna Ribeiro Porto, Maitê Cardoso Coelho da Silva, Pedro Nacib Jorge

**Affiliations:** 1 Instituto Reprocon, Campo Grande, MS, Brasil; 2 Faculdade de Medicina Veterinária e Zootecnia, Universidade de São Paulo, São Paulo, SP, Brasil; 3 NEX-No Extinction, Corumbá de Goiás, GO, Brasil; 4 Faculdade de Medicina Veterinária, Centro Universitário de Brasília, Brasília, DF, Brasil; 5 Instituto de Biociências, Universidade Federal de Mato Grosso do Sul, Campo Grande, MS, Brasil; 6 Faculdade de Medicina Veterinária e Zootecnia, Universidade Federal de Mato Grosso do Sul, Campo Grande, MS, Brasil

**Keywords:** laparoscopic evaluation, biobanking, ovaries, conservation, Caatinga biome

## Abstract

The *in situ* population of jaguars in the Caatinga is less than 250 individuals, subdivided into five subpopulations, and is classified as endangered regarding its risk of extinction. Luisa, a 15-year-old female weighing 36 kg, was the last known *ex situ* jaguar from this biome. Her reproductive evaluation is detailed in this manuscript. Luisa was subjected to both a clinical and laparoscopic evaluation of her reproductive system. After 45 days of reproductive investigation, she died unexpectedly, and skin fragments were taken to establish the *postmortem* fibroblast lineage. At the clinical evaluation, Luisa had small, undeveloped mammary gland and a small vulva, characteristic of a nulliparous female, with no mammary gland nodules, edema, or abnormal masses. By laparoscopy, normal-appearing bladder and bowel loops were observed, as were uterine horns with standard color, shape, and length with no striae. Ovaries and uterine horns seem free of fibrinous adhesions. Both ovaries showed a yellowish color, a fibrous consistency, a decreased size (atrophied), and no follicles, hemorrhagic corpus, corpus luteum, luteal scars, or other abnormal structures. We may assume that this jaguar female was infertile based on Luisa's mature age and the absence of birthing or ovarian activity signs. The harsh conditions of the Caatinga biome, which included low food availability and frequent conflicts with humans, may have impacted both the pregnancy and lactation of Luisa’s mother and her development after birth.

## Introduction

Jaguar is widespread in five of the six Brazilian biomes and is currently classified as “endangered” related to its risk of extinction in the Caatinga biome (ICMBio, 2018), where its population is estimated to number less than 250 individuals and is separated into five isolated subpopulations (Paula et al., 2012). Therefore, inbreeding poses a threat (Araújo et al., 2021) to the long-term survival of the species in the biome.

In accordance with the One Conservation approach (Pizzutto et al., 2021; Yarto-Jaramillo et al., 2022), it is essential to develop an *ex situ* security population of jaguars from the Caatinga in addition to *in situ* and anthropic actions. Luisa, an estimated 15-year-old female, was the sole *ex situ* jaguar known worldwide to have sprung from the Caatinga.

After conflicts with livestock, Luisa was imprisoned in a cave in the municipality of Sento Sé (Bahia, Brazil), near the Boqueirão da Onça National Park, in April 2019. Luisa was still alive after 22 days and was rescued by the Amigo das Onças project. The female was released with a GPS collar two months after *ex situ* care. However, in February 2020, following fresh clashes with livestock, she was imprisoned in a cave once more and relocated permanently to an *ex situ* environment, where assisted reproduction would be attempted. One year later, in February 2021, Luisa was finally relocated to NEX-No Extinction.

Therefore, the purpose of the clinical evaluation was to evaluate Luisa's reproductive health and to document the findings of this emblematic female in order to preserve the species in the Caatinga.

## Materials and methods

The reproductive evaluation was performed on 04^th^ October 2021, in Brazil at the NEX-No Extinction, a Scientific Breeding Center (Corumbá de Goiás, GO, Brazil; 15°51'32.3”S 48°28'28”W), where the female stayed from the time of her relocation until the time of her death. The study reports the outcomes of the clinical examination conducted by veterinarians. These examinations are regarded as a zootechnical practices and are not subject to regulation by Brazilian Federal Law no. 11,794/2008, as they are unrelated to teaching or scientific research activities (MCTIC, 2023). Therefore, approval from an Ethics Committee on the Use of Animals is not required. In accordance with ethical recommendations for assisted reproduction of wild animals (Pizzutto and Jorge-Neto, 2023), the use and treatment of experimental animals were in compliance with Brazilian laws, guidelines, and policies on animal welfare. Under the protocol AEA074D, the genetic heritage accessions were recorded in Brazilian National System for the Management of Genetic Heritage and Associated Traditional Knowledge (SISGEN).

### Technical procedure for reproductive evaluation

Chemical restraint was administered with anesthetic darts fired by a blowpipe and containing ketamine (5 mg/kg; im) and medetomidine (0.1 mg/kg; im) (Araujo et al., 2018), followed by anesthetic induction with propofol (4 mg/kg; iv). Periglottic anesthesia was also administered with lidocaine for intubation, and 1% isoflurane was used to maintain anesthesia. During the entire procedure, veterinarians monitored the animal's physiological parameters, plethysmography curve, anesthetic plane, and degree of myorelaxation while administering lactated Ringer's solution therapy. Blood was drawn for a complete blood count, smears for hemoparasites, and biochemical analysis (creatinine, alkaline phosphatase, pyruvate transaminase, and urea). After the procedure, anesthesia was reversed using atipamezole (0.75 mg/kg; im), and meloxicam (0.05mg/kg; im) was used for analgesia.

Mammary glands were palpated to determine the presence of nodules, edema, or tumors, as well as their progression, during a thorough clinical examination that focused mainly on the reproductive system. It was also investigated the vulva's anatomical appearance, alterations, and fluid or secretion presence.

The female was then positioned in Trendelenburg position (45° angle) on a laparoscopy table (Novagen, Brazil) for laparoscopic explorations. Using a 5 mm laparoscope and grasping forceps, both ovaries were examined for the presence of structures (follicles, hemorrhagic corpus, corpus luteum, and abnormal structures), and the uterus was examined for the presence of striae indicating past pregnancies or abnormal structures. Our study team based all laparoscopic procedures on earlier works performed on jaguars (Jorge et al., 2018; Jorge-Neto et al., 2023; Jorge-Neto et al., 2020a).

### Establishment of a postmortem fibroblast lineage

Due to acute kidney failure, Luisa died suddenly after 45 days of the reproductive examination. Skin fragments and ovaries were collected hours after the animal was pronounced dead and prior to the *postmortem* investigation. The technique entails shaving a portion of the inner groin, washing the region with 70% alcohol, and allowing it to air-dry. With a scalpel, a 1 cm x 1 cm square chunk of skin was removed. Two fragments were obtained and put in a buffer solution (Dulbecco’s phosphate-buffered saline without CaCl_2_ and MgCl_2_). The uterus and ovaries were exposed by an incision in the *linea alba* and peritoneum with a scalpel directly caudal to the umbilicus. The ovaries were extracted and placed into the buffer solution. Biopsy samples were transported in a sterile plastic container at 5°C. To prevent sample dehydration, the solution volume should be sufficient to cover the entire tissue sample(s). For safe transport and to prevent the sample from freezing due to direct contact with the coolant, the plastic container must be wrapped in paper and stored in a plastic bag.

Tissue samples were sent to the commercial company Bio Cell Terapia Celular (Brasilia, DF, Brazil; 15°50'38.9”S 48°01'45.7”W), where they were prepared for cell isolation. Finally, the fibroblasts were cryopreserved and stored in the biobank of Reprocon Institute.

## Results

**Figure 1:**
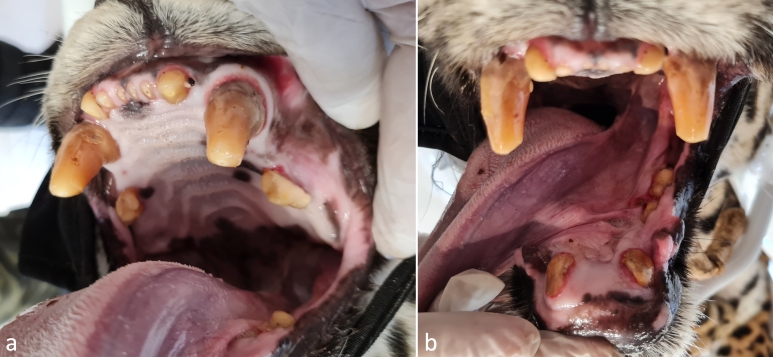
Visible wear on upper jaw teeth (a) and lower jaw broken teeth (b).

Luisa weighed 36 kg. Regarding the dentition (Figure 1), the lower jaw lacked incisors and had thoroughly fractured canines at their base, revealing just the cervical third and one missing premolar on each side. The canines and incisors had significant enamel wear in the upper jaw, exposing the dentin. It possessed a single premolar on each side. The female also exhibited receding gums.

**Figure 2:**
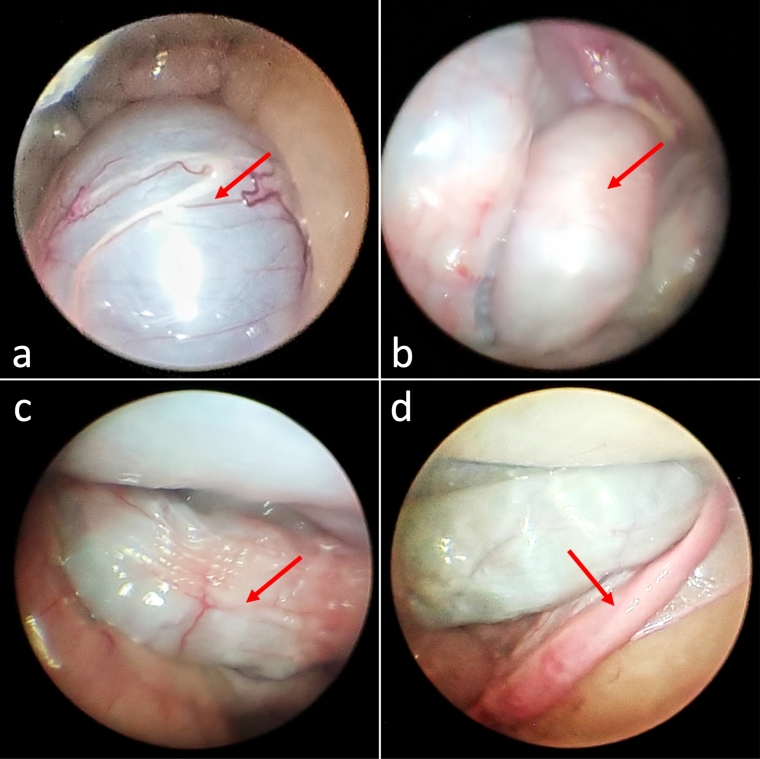
Evaluation by laparoscopy of Luisa: (a) urinary bladder, (b) intestine loop, (c) intestine loop with fecal content, and (d) right uterine horn.

**Figure 3:**
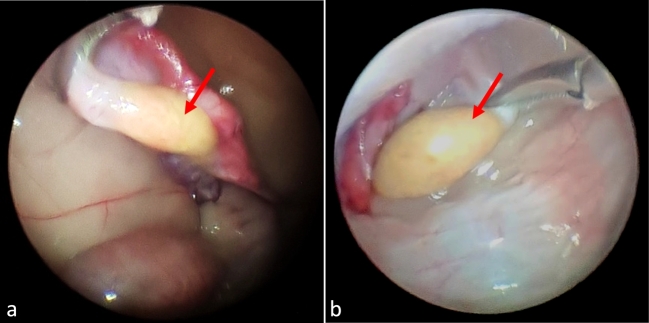
Evaluation by laparoscopy of Luisa: (a) right ovary, and (b) left ovary.

The following external visual characteristics were noted: mammary glands that were small and underdeveloped, indicative of a nulliparous female; absence of mammary gland nodules, edema, or abnormal masses; and small vulva. The following internal visual characteristics were observed (Figure 2 and Figure 3): normal bladder and bowel loops; uterine horns with standard color, shape, and length with the absence of striae on the uterine horns, suggesting a nulliparous female; ovaries and uterine horns without fibrinous adhesions; both ovaries had a yellowish hue, a fibrous consistency, and no follicles, corpus hemorrhagicum, corpus luteum, luteal scars, or other abnormal structures.

At the time of her laparoscopic evaluation, Luisa's body score was low, and her coat was drab and bristly. She exhibited normophagia, regular water consumption, normal urine and feces, and expected, active, and responsive ambulation. In the last days of her life, she was highly prostrated, had corneal opacity (without sparkles), was dehydrated, and was hypoxic, but she drank normal quantities of water and had normal urination, despite having urinary incontinence. Dull, disheveled fur and a small number of normal-appearing stools. Urine with a highly pungent odor.

## Discussion

There are genetic differences among *in situ* jaguars from the five biomes in Brazil where they are found. Specifically, jaguars located in the Caatinga biome were found to be the most genetically distant from those in other biomes (Lorenzana et al., 2022 forthcoming). Despite these genetic distinctions, Lorenzana's study failed to identify population substructures in their sampled populations.

Despite significant phenotypic variation across jaguars inhabiting distinct biomes, the species is not divided into subspecies (Eizirik et al., 2001); whereas a female jaguar in the Caatinga weighs approximately 30 kg (Araújo, unpublished), a female jaguar in the Pantanal can weigh up to 95 kg (Morato et al., 2018; Reprocon, unpublished) and in the Amazonia they average 40 to 55 kg (Morato et al., 2018; Palmeira and Trinca, 2012). Therefore, when considering *ex situ* mating, it is essential to take into account the specific biome and phenotypic similarities among jaguars to make informed decisions.

As there is no *ex situ* population and the species is endangered related to its risk of local extinction, the situation of the jaguar in the Caatinga is quite alarming. This biome requires special attention due to its extractive exploitation by the local population, who frequently rely on hunting for livelihood, and its inherently unfriendly environment to native animals like the jaguar, who traverse long distances in search of food and water (Martins and Jorge-Neto, 2022). Less than 250 jaguars are separated into five isolated subpopulations in the Caatinga (Paula et al., 2012). The dispersion between these subpopulations should occur in order to maintain a large outbred population (Björklund, 2003) and avoid possible reproductive performance issues caused by the decline in heterozygosity levels, as occurred in the isolated population of Ngorongoro lions (Packer et al., 1991).

To conserve jaguars in the Caatinga, immediate actions focused on long-term conservation and pursuing the One Conservation strategies are required. Therefore, in addition to implementing ecological corridors to connect subpopulations of jaguars in the Caatinga (Morato et al., 2014) and working with acceptance – or changing human communities' perceptions of jaguars (Martins et al., 2021, 2022; Santos et al., 2007), it is required to establish a viable *ex situ* population of Caatinga jaguars. Even temporarily, it is vital to take free-living jaguars from the Caatinga to captivity. *Ex situ* reproduction of free-ranging jaguars has been demonstrated to be viable, even when the adult female is removed to captivity as an adult and under severe stress (Luczinski et al., 2023).

Due to dentin deposition in the pulp chamber, the estimated Luisa’s age is greater than 15 years, resulting in a pretty narrow chamber. All of the teeth showed a brownish hue, consistent with the production of tertiary (or reparative) dentin as a result of the irritative processes of the odontoblasts caused by age-related wear. Even though jaguars are considered senile at age 15, they can still reproduce. Using a GPS collar, the Reprocon Institute tracked a 17-year-old female and her offspring in the Pantanal (unpublished), and the oldest reliably recorded *ex situ* female giving birth was 20.5 years old (AZA Jaguar Species Survival Plan, 2016). In the closely related leopard (*Panthera pardus*), Balme et al. (2013) report a female giving birth at 16.3 years of age. Jaguar presents year-round estrus ( Jimenez Gonzalez et al., 2017; Morato et al., 2004) with a 38.28 ± 2.52 days oestrous cycle (Viau et al., 2020); however, neither estrus signals nor pre-copulatory vocalization (Jorge-Neto et al., 2018) was ever observed during Luisa's ten months at NEX. In addition, the clinical examination revealed small, underdeveloped mammary gland and a standard small vulva, indicative of a nulliparous female.

Laparoscopy was chosen for the evaluation because it is minimally invasive and allows accurate visualization of the ovarian and uterine structure, which was not feasible with ultrasonography in jaguars (Jorge-Neto et al., 2023). While healthy jaguar ovaries (Jorge-Neto et al., 2023; Jorge-Neto et al., 2020a) are pink to bright red in color, Luisa's ovaries were yellow with atrophic and fibrotic characteristics (Figure 2). Compared to other jaguars (Jorge-Neto et al., 2023), the apparent atrophy and fibrosis of the ovaries indicate the absence of cyclic ovarian activity. In addition to the absence of uterine striae, she had a diminutive size of the mammary gland and vulva. These traits are inconsistent with prior pregnancies, leading to the presumption that this female was nulliparous, even though her age could be estimated to be over 15 years old.

There is little knowledge of the causes of infertility in *in situ* female wild cats other than mating difficulties, ovulation disorders, uterine pathology, hormonal problems, or viral infections. Other possible causes of infertility in domestic cats include malnutrition, chromosomal or genetic issues (Fontbonne, 2022; Fontbonne et al., 2020), and viral infections such as feline herpesvirus 1, feline leukemia virus, feline panleukopenia virus, and feline infectious peritonitis virus (Foster, 2017). After quarantine, however, Luisa tested negative for such viral diseases before being referred to NEX. Although the domestic cat is distant from jaguars in terms of phylogenetic relations among felid species (Johnson et al., 2006), it is undeniably an important model (Silva et al., 2021). It is impossible to say, however, it is wise to presume that the Caatinga biome is challenging for an apex predator. The likelihood that Luisa suffered from malnutrition throughout her formative years significantly impacted her gonadal development. Lack of critical nutrients such as taurine, arachidonic acid, copper, and polyunsaturated fatty acids can affect the reproductive performance of domestic cats (Fontbonne, 2022). Likewise, experimental malnutrition causes retarded sexual development, cessation of estrous cycles, and ovarian atrophy in rats (Nakanishi et al., 1976). The effects of maternal malnutrition during pregnancy and/or lactation can also extend to the offspring. Léonhardt et al. (2003) discovered that exposure to malnutrition during pregnancy severely affected the development of the gonads in the offspring of both sexes from weaning and possibly beyond.

Inbreeding in pedigreed domestic cats is frequently cited as a reason for poor reproductive performance (Fontbonne, 2022; Fontbonne et al., 2020). In cheetahs (*Acinonyx jubatus*), genetic homogeneity did not appear to affect reproduction (Merola, 1994), while it had a negative impact on lion (*Panthera leo*) testes (Munson et al., 1996). The loss of genetic variability would be a testable hypothesis given the low population density and isolation of subpopulations of the Caatinga jaguar. Nevertheless, the genetic analyses performed by another research group (unpublished data) to date do not indicate^1^ that Luisa has high levels of inbreeding. Her genetic diversity is similar to what is found in most jaguars^1^ from other Biomes.

A possible sterile free-living jaguar male has been found in Pantanal, and the potential impact on the local population has been questioned (Jorge-Neto et al., 2020b). However, the discovery of an adult female jaguar that has not given birth in the wild is clearly alarming, particularly in a biome where the species is endangered regarding its risk of extinction. Whether due to poor nutrition or a lack of mating opportunities, it is likely that this jaguar did not fulfill its role in perpetuating the species, and cloning is the only option for saving its important genetics.

Luisa was set to have a new laparoscopic assessment with hormonal stimulation of the ovaries followed by a uterine and ovarian biopsy, but she died unexpectedly beforehand. On this occasion, ovaries with follicles would be punched in search of oocytes. If there were no responses to the hormonal stimulus, one or both ovaries would be removed for histological analysis to support the infertility diagnosis.

The autopsy report's conclusion was nephrotic syndrome. Chronic kidney disease (CKD) is a progressive and irreversible morphofunctional condition of one or both kidneys, characterized by an insidious progression, heterogeneous etiology (mostly unidentifiable), and a progression lasting longer than three months. In contrast to canines and humans, feline CKD is primarily characterized by tubular pathology; primary glomerular diseases are uncommon in this species. Tubulointerstitial nephritis is a progressive and multifactorial syndrome that culminates in chronic intrarenal inflammation associated with fibrosis (Breshears and Confer, 2017). Its pathophysiological mechanisms remain unknown. Before the cadaver was sent for necropsy, the ovaries were removed and sent along with the tissue to the laboratory in an attempt to obtain oocytes; however, as they arrived already putrefied, they could not be used. Therefore, histopathological examination was not feasible on these ovaries.

In order to avoid scientific prevarication, we disclose our findings on the last *ex situ* jaguar from the Caatinga, so documenting this scenario and contributing to the generation of knowledge about this species can serve as the basis for future research.

## Conclusion

In conjunction with Luisa's adult age and the absence of any signs of parturition or ovarian activity, we can conclude that this jaguar female was infertile. While genetic analysis has not yet revealed any indications of inbreeding that could explain this infertility, malnutrition is a possibility. The severe conditions of the Caatinga biome, including limited food availability and frequent conflicts with humans, may have affected both the pregnant/lactation mother when Luisa was born as well as Luisa's development after birth.

## Footnotes

1 Sartor and Eizirik (2023) personal communication of ongoing specific research conducted by another group).
